# Splenic function post radiation therapy in childhood cancer survivors

**DOI:** 10.1016/j.ctro.2025.100961

**Published:** 2025-04-13

**Authors:** Joy Fulbright, Kyla Alsman, Ashley K. Sherman, Kris Laurence, Becky Lowry

**Affiliations:** a Division of Hematology/Oncology/Bone Marrow Transplant, Children’s Mercy, 2401 Gillham Road, Kansas City, MO 64108, USA; b University of Kansas Health System, 2000 Olathe Blvd, Kansas City, KS 66160, USA; c Biostatistics & Epidemiology Core, Division of Health Services & Outcomes Research, Children’s Mercy Kansas City, Kansas City, MO, USA; d Children’s Mercy Research Institute, 2401 Gillham Road, Kansas City, MO 64108, USA; e Department of Internal Medicine, Kansas University Medical Center, 3901 Rainbow Boulevard, Kansas City, KS 66160, USA

**Keywords:** Childhood cancer survivor, Splenic function, PIT count, Radiation, Infection

## Abstract

Study evaluated splenic function in childhood cancer survivors (CCS) who had received radiation to the spleen utilizing pRBC PIT (vesiculated erythrocytes) count compared to a non-radiated control group. Those exposed to < 20 Gy of radiation had PIT counts not significantly different from the control group. There was trend for higher pRBC PIT count in those exposed to higher doses of radiation.

## Highlights

• Elevated PIT count correlates with splenic dysfunction.
• Trend for higher splenic doses of radiation to increase PIT count in CCS.
• PIT count may be marker to evaluate CCS for splenic dysfunction.

## Introduction

Childhood cancer survivors (CCS) who have intraabdominal tumors such as Wilm’s tumor, other renal cancers, Hodgkin’s lymphoma involving the spleen or intrabdominal sarcomas may receive treatment with abdominal radiation that impacts the spleen. Splenic field radiation has been compared to splenectomy for the associated increase in infection-related health risk [1]. The spleen plays an important role in the immune system by providing defense against bacteria using the iron metabolism of macrophages, development and storage of B and T lymphocytes, production of immune mediators involved in clearance of bacteria and phagocytosis of circulating microorganisms and immune complexes [2]. People who have had their spleen removed are known to have an increased risk of potentially life-threatening infections specifically from encapsulated organisms- Streptococcus pneumonia, Haemophilus influenzae and Neisseria meningitis [3]. Vaccines against these organisms are a critical component of the care plan for patients who have undergone splenectomy [4], [3]. Penicillin prophylaxis is recommended especially during the first 2 years post splenectomy and in those under the age of 5 [5], [3].

The guidelines on how to manage CCS who have received radiation impacting the spleen are vague. One recent Children’s Oncology Group (COG) study for Hodgkin Lymphoma patients recommended “all patients undergoing splenectomy or splenic irradiation should be immunized with polyvalent pneumococcal, HIB and meningococcal vaccine. Irradiated spleens left in situ may not be fully functional.” But there are no further recommendations on longitudinal care plans for these patients as it relates to antibiotic prophylaxis or targeted vaccinations. Weiner et al. evaluated PIT count in patients with advanced Hodgkin’s disease [6]. Vacuolated erythrocytes count or PIT count is an assay that determines the number or erythrocytes that contain pits when studied with electron microscopy. The pits are vacuoles beneath or attached to the plasma membrane. An elevated PIT count indicates that the spleen is not functioning properly. In patients with sickle cell disease a PIT count of ≥3.5 percent was associated with a decreased or absent uptake on radionuclide spleen scans [7]. A more recent study in patients with sickle cell disease demonstrated that normal splenic function is predicted by PIT count of ≤1.2 and a PIT count ≥4.5 is correlated with absent function [8]. In this study by Rogers no child with normal splenic function measured by 99 m Tc sulfur-colloid liver-spleen scan had an elevation of PIT count >3.5 %. Weiner et al. evaluated patients who received chemotherapy alone vs chemotherapy plus radiation to the spleen (21 Gy) or chemotherapy plus splenectomy. Patients in the first two groups had a mildly elevated PIT count of 3.2 % and 3.8 % (not statistically different) compared to the splenectomy group who had a count of 36.7 %. The conclusion from this study was that low dose radiation did not decrease splenic function any more than just receiving chemotherapy. Based on the data from Rogers et al. study both of these groups could have had impaired splenic function given PIT counts averaged to be ≥1.2. Coleman et al. also evaluated splenic function in patients post radiation for Hodgkin’s disease. They demonstrated that when patients received >40 Gy radiation they had a mean PIT count of 13 % [9]. In normal subjects mean PIT count was 0.9 % and 33.7 % in those post splenectomy. Both papers evaluated survivors treated over 40 years ago.

Weil reviewed infection related late mortality in 20,805 five-year CCS diagnosed from 1970 to 1999 [1]. There was an increased risk of sepsis in patients who received splenectomy and in those with no splenectomy but who received left upper abdominal radiation with risk increasing based on dose of radiation received. Survivors who received just 10–19.9 Gy had an increased absolute excess risk of infection related mortality compared to those who did not receive radiation or splenectomy. This was a retrospective chart review that was not able to directly correlate splenic function with dose of radiation but is suggestive that cancers survivors exposed to left upper quadrant radiotherapy, even at lower doses, are at increased risk for functional asplenia.

Due to the paucity of recent data on this area and conflicting results in the literature, we evaluated splenic function in our survivors that received radiation to a field that included the spleen using PRBC PIT counts.

## Methods

This was a multi-institutional prospective cohort study. IRB (Internal Review Board) approval was obtained through Children’s Mercy IRB (approved 1/21/2019 SITE00000536) with The University of Kansas Health System relying on Children’s Mercy IRB. Study participants were recruited from patients attending the childhood cancer survivorship clinics at each of the two study sites and enrolled from March of 2019 until August of 2022. Written informed consent was received from survivors 18 years and older or from the legal guardian of those under 18 with assent given by the survivor if they were age 7 or older. A control group included CCS who did not receive any radiation therapy or splenectomy, but did receive chemotherapy for Hodgkin’s lymphoma or Wilms’s tumor. The recruited treatment group received radiation therapy that included the spleen along with chemotherapy. The radiation dose was the prescribed dose of radiation. CCS were excluded if they did not have treatment records available for review, were less than 2 years from end of therapy, or received autologous or allogeneic stem cell transplant.

Participants were screened for eligibility prior to their clinic appointment and consented prior to blood draw. If no labs were scheduled to be drawn, study consent included risk of lab draw as an additional risk.

Peripheral RBC PIT count was measured as a surrogate marker of splenic function in our study population as it does not require radiation exposure and could be obtained at the same time as other labs were being drawn. RBC PIT count was a send out test to the clinically available laboratory at the time of study development, Cincinnati Children’s hospital, and measured manually by microscopy using differential interference contrast on a light microscope with Nomarski optics. Lab had to be drawn prior to 1400 on Mon.-Thurs. so could be sent overnight and processed the next day.

Demographic data was captured and stored in a REDCap (Research Electronic Data Capture) database including the following: cancer diagnosis, age at time of diagnosis, dose of radiation received, radiation field (ie left flank, versus whole abdominal), time from completion of chemotherapy and radiation therapy, and age of patient at time of PIT draw, was collected and stored in a REDCap database. Treatment and diagnosis were reviewed and participants were assigned an Intensity of Treatment Rating score (ITR-3) [10]. Additionally, self-reported immunization records and infection history were gathered since completion of cancer therapy.

Descriptive statistics such as medians, interquartile ranges (IQR) and proportions were used to summarize the data. Fisher’s Exact and Wilcoxon Rank Sum tests were used to look for differences in groups. SAS version 9.4 (SAS Institute Inc., Cary, NC) was used for all analyses.

## Results

**Fig. 1:**
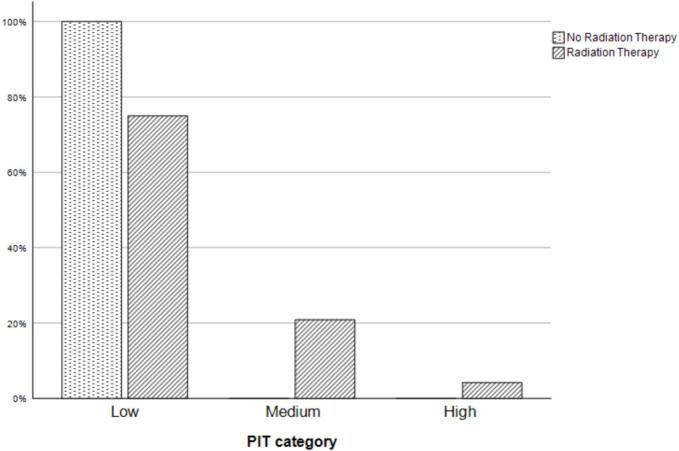
Percentage of participants receiving no radiation vs radiation in each PIT risk category.

**Fig. 2:**
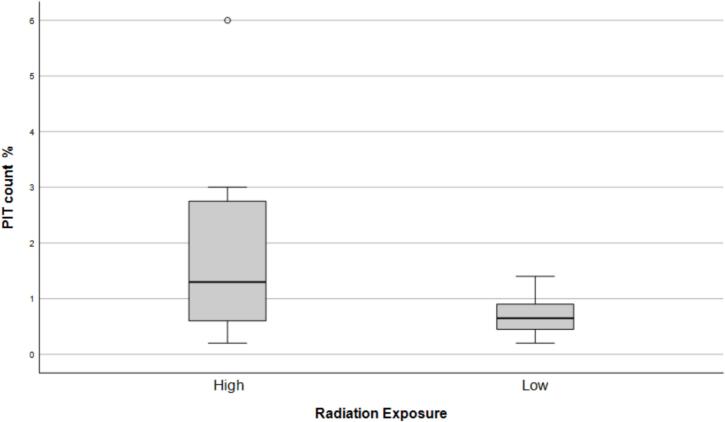
PRBC PIT count percentage in those exposed to high radiation (≥20 Gy) vs those exposed to low radiation (<20 Gy).

We enrolled a total of 34 survivors with 30 completing the study by obtaining PIT count. We excluded the 4 that did not present to the lab to have PIT count drawn from our analysis. Median age at cancer diagnosis was 4 years (IQR 2.3–9.8). Six were in the control group (no radiation), sixteen in <20 Gray (range 10–19.8) and seven in the ≥20 Gray of radiation (Table 1). All of the low-risk radiation patients received either whole abdominal or flank radiation at a median prescribed dose of 10.5/10.8 Gy. In the high-risk group four patients received whole abdominal radiation and two received mantle radiation to 21 and 30 Gy. Median dose of radiation was 24 Gy for the high-risk group. Of those who received radiation therapy the median time from the completion of radiation to collection of PIT count was 112 months (IQR 75–164). We first grouped PIT count into 3 categories and looked at the relationship to radiation. We stratified them into 3 risk groups based the results from Pearson et al. and Rogers et al. [7], [8] low risk group < 1.2; intermediate group 1.2–3.4 and high-risk group 3.5 or higher. The three groups also allowed us to evaluate if there was a subtle difference in PIT count that perhaps explained Weil’s results. Of those who did not receive radiation a 100 % were in the lowest PIT count group (<1.2). Of those who received radiation 75 % (N 18) were in the lowest PIT count group, 21 % (N 5) in the middle (1.3–3.4) and 4 % (N 1) were in the highest (≥3.5) Fig. 1. This difference in proportion however was not statistically significant (p = 0.642). Next, we looked at the relationship between the amount of radiation (for those who got radiation) and PIT count. Eighty-eight percent (N 14) of the low radiation group were in the low PIT count group with the remaining 12 % (N 2) in the middle PIT count group. The high radiation group had 50 % (N 4) in the lowest PIT count group, 38 % (N 3) in the middle group and 14 % (N 1) in the highest group (p = 0.045) Fig. 2. We also looked at the relationship between radiation group (low vs. high) and the raw PIT count variable and found no significant difference [low group median 0.65 (IQR 0.45–0.90) and high group median 1.30 (IQR 0.50–3.0), p = 0.159.

**Table 1:** Demographics

	Controls n = 6	Low dose radiation (<20 Gy) n = 16	High dose radiation (≥20 Gy) n = 7
Age at diagnosis (months), median (IQR)	151.5 (76.5–170.8)	39.6 (20.0–56.6)	42.0 (34.6–167.6)
Intensity of treatment score, n (%)			
2	4 (66.7)	1 (6.3)	2 (28.6)
3	2 (33.3)	15 (93.8)	4 (66.7)
4	0 (0)	0 (0)	1 (16.7)
Time from radiation to PIT count (months), median (IQR)		108 (74.5–125.5)	156 (93–234)
Dose of radiation (Gy), median (IQR)		10.7 (10.5–10.8)	24 (21–36)
Field of radiation		10 left flank 6 whole abdomen	5 whole abdomen 2 mantle

Finally, we looked at the amount of radiation (high vs. low) and time from end of radiation to data collection. The low radiation group had a median time of 108 months (IQR 74.5–125.5) and the high radiation group had a median time of 156 months (IQR 93–234) and this difference was not significantly different (p = 0.116). This was evaluated as hypothesized that time from radiation may affect splenic function.

Regarding infections post therapy only 1 participant noted to be diagnosed with pneumonia (PIT count of 0.6 %) and none with meningitis. There was also only one recorded hospital admission for infection, MRSA of the hand.

## Discussion/conclusion

Though sample size was lower than recruitment goal we noted that those who did not receive radiation all had a PIT count in the lowest quartile. Those at most risk for having a higher PIT count/ splenic dysfunction were those who received higher than 20 Gray of radiation, though only 1 of these patients had a PIT count that is in the range considered to be asplenic. Based on data from sickle cell population 5 of our survivors had intermediate risk PIT PRBC counts. Therefore, they were not asplenic, but spleen was likely not fully functioning.

To further evaluate effect of time from radiation on PIT count a longitudinal study would be beneficial where measure serial PIT counts from same patient population over time to see if PIT count changes. Future study with a larger cohort would also add to our knowledge on how to educate patients and families. The Weil et al. data suggests that lower exposures may affect splenic function. Our study also had patients receiving less than 20 Gy of radiation in the intermediate PIT count group indicating some splenic dysfunction. All of the patients in this study received radiation therapy in year 2000 or later except for one patient. Radiation techniques have improved overtime and there may be less splenic exposure using these newer techniques.

In our evaluation of patients, we did not have access to details of the patient’s radiation plans to extract data such as Dmean/Dmax to the spleen. In general, when CCS are followed for survivorship the most information clinicians have access to is field of radiation and dose of radiation. For this reason, when survivorship guidelines are developed, they are based on this limited radiation exposure information so that they can be applied in real world settings. More granular detail regarding how much each organ at risk was exposed to could lead to more specific guidance to each survivor on risk.

Sample size in our study was smaller than our goal of 50. It was affected by COVID pandemic occurring shortly after opening the study (many survivors switched to being seen via telehealth with local labs). Time frame that the PIT count could be collected also influenced recruitment. PIT count was a send out lab that could only be collected Mon.-Thurs. prior to 1400. Many of our survivors are evaluated in clinic on Friday afternoon. Therefore, to be on study had to return for this lab draw. Another limitation of the study was that the infections a was self-reported so recall bias may affect the outcomes reported.

Albeit small, this study does support consideration of obtaining a PIT count in survivors who are at higher risk of splenic dysfunction (i.e., > 20 gy of radiation in a field that includes the spleen) as a simple way to screen for splenic dysfunction. This will allow providers to impart education to survivors and families regarding risk of infections. Also, will help us educate and encourage at risk population to obtain protective vaccines for pneumococcal, haemophilus influenza and meningococcal. An area we could improve overall health of cancer survivors is to encourage them to receive recommended immunizations. Data demonstrated that provider recommendation to receive vaccinations post therapy increases the likelihood that a CCS will restart vaccinations [11]. There is more data that is evolving on CCS having immune dysregulation and increase risk of infections post therapy, [12] so it is extremely important that CCS are protected as much as possible from infections.

## Author contributions

Joy Fulbright and Becky Lowry contributed to the design of the trial. Ashley Sherman contributed to power calculations, statistical plan and statistical analysis of the data. Kyla Alsman, Becky Lowry, Joy Fulbright, and Kris Laurence worked on IRB submission, screened patients for the trial, enrolled patients on to the trial and performed data collection. Joy Fulbright wrote the first draft and all authors reviewed and edited the manuscript.

## Funding

This work was supported by the Children’s Mercy Tom Keaveny Endowed Fund for Pediatric Cancer Research and the Masonic Cancer Alliance grant.

## Data availability

The datasets generated during and/or analyzed during the current study are available from the corresponding author on reasonable request.

## Declaration of competing interest

The authors declare that they have no known competing financial interests or personal relationships that could have appeared to influence the work reported in this paper.

## References

[1] Weil B.R., Madenci A.L., Liu Q., Howell R.M., Gibson T.M., Yasui Y. (2018). Late infection-related mortality in asplenic survivors of childhood cancer: a report from the childhood cancer survivor study. J Clin Oncol.

[2] de Porto A.P., Lammers A.J., Bennink R.J., ten Berge I.J., Speelman P., Hoekstra J.B. (2010). Assessment of splenic function. Eur J Clin Microbiol Infect Dis.

[3] Guri A., Ben-Ami T. (2024). Updated Recommendations on the prevention and treatment of infections in children with asplenia/hyposplenism. J Pediatr Hematol Oncol.

[4] Red Book. 31st ed. United States of America: American Academy of Pediatrics; 2018.

[5] Rubin L.G., Schaffner W. (2014). Clinical practice. Care of the asplenic patient. N Engl J Med.

[6] Weiner M.A., Landmann R.G., DeParedes L., Leventhal B.G. (1995). Vesiculated erythrocytes as a determination of splenic reticuloendothelial function in pediatric patients with Hodgkin's disease. J Pediatr Hematol Oncol.

[7] Pearson H.A., Gallagher D., Chilcote R., Sullivan E., Wilimas J., Espeland M. (1985). Developmental pattern of splenic dysfunction in sickle cell disorders. Pediatrics.

[8] Rogers Z.R., Wang W.C., Luo Z., Iyer R.V., Shalaby-Rana E., Dertinger S.D. (2011). Biomarkers of splenic function in infants with sickle cell anemia: baseline data from the BABY HUG Trial. Blood.

[9] Coleman C.N., McDougall I.R., Dailey M.O., Ager P., Bush S., Kaplan H.S. (1982). Functional hyposplenia after splenic irradiation for Hodgkin's disease. Ann Intern Med.

[10] Kazak A.E., Hocking M.C., Ittenbach R.F., Meadows A.T., Hobbie W., DeRosa B.W. (2012). A revision of the intensity of treatment rating scale: classifying the intensity of pediatric cancer treatment. Pediatr Blood Cancer.

[11] Warner E.L., Vaca Lopez P.L., Kepka D., Mann K., Kaddas H.K., Fair D. (2020). Influence of provider recommendations to restart vaccines after childhood cancer on caregiver intention to vaccinate. J Cancer Surviv.

[12] Chehab L., Doody D.R., Esbenshade A.J., Guilcher G.M.T., Dvorak C.C., Fisher B.T. (2023). A population-based study of the long-term risk of infections associated with hospitalization in childhood cancer survivors. J Clin Oncol.

